# What is the easier and more reliable dose calculation for *iv* Phenytoin in children at risk of developing convulsive status epilepticus, 18 mg/kg or 20 mg/kg?

**DOI:** 10.1186/1471-2431-13-60

**Published:** 2013-04-21

**Authors:** Manish Prasad, Priya Shenton, Sanne Dietz, Vivek Saroha, William P Whitehouse

**Affiliations:** 1 Department of Paediatric Neurology, Nottingham Children’s Hospital, Nottingham University Hospitals NHS Trust, Nottingham, UK; 2 School of Clinical Sciences, University of Nottingham, Nottingham, UK

## Abstract

**Background:**

With the Convulsive Status Guidelines due for renewal, we wondered if a phenytoin dose of ‘20 mg/kg’ would be easier to calculate correctly and therefore safer than the current ‘18 mg/kg’. An educational exercise in dose calculation was therefore undertaken to assess ease of calculation.

**Method:**

A standard question paper was prepared, comprising five clinical scenarios with children of varying ages and estimated body weights. Medical students, trainee doctors at registrar and senior house officer level, and consultant paediatricians were asked to complete the exercise, in private, by one of two medical students (SD, PS). Calculations were done with and without a calculator, for 18 mg/kg and for 20 mg/kg in randomised order. Speed and errors (greater than 10%) were determined. The data analysis was performed using SPSS version 18.

**Results:**

All answered all 20 scenarios, giving a total of 300 answers per doctor/student group, and 300 answers per type of calculation. When comparing the 2 doses, the numbers of errors more than 10% were significantly less in 20 mg/kg dose (0.33%) as compared to the 18 mg/kg dose (9.3%) (p<0.0001). Speed off calculation was significantly decreased in 20 mg/kg dose when compared with 18 mg/kg dose, with (p<0.001) or without (p<0.0001) the calculator. Speed was more than halved and errors were much less frequent by using a calculator, for the 18 mg/kg dose but no difference with or without the calculator for 20 mg/kg dose.

**Conclusion:**

We recommend that the future guidelines should suggest iv Phenytoin at 20 mg/kg rather than 18 mg/kg. This will make the calculation easier and reduce the risk of significant errors.

## Background

With the convulsive status epilepticus in children guideline due for renewal, we wondered if a Phenytoin dose of ‘20 mg/kg’ would be easier to calculate correctly and therefore safer than the previously recommended ‘18 mg/kg’ dose. An educational exercise in dose calculation was therefore undertaken.

Convulsive status epilepticus (CSE) is defined as a continuous or recurrent convulsive seizure with loss of consciousness lasting 30 minutes or more, or a cluster of repeated convulsions during which consciousness is not regained, lasting 30 minutes or more [1]. CSE in childhood constitutes a medical emergency as it is a life threatening condition with serious risk of neurological squelae [2]. In addition, the longer the duration of the episode, the more difficult it is to terminate [3].

Data from epidemiological studies suggest that four to eight children per 1000 may be expected to experience an episode of CSE before the age of 15 years [4], and in children with first seizures, 12% present with CSE as their first unprovoked seizure [5]. CSE in children has a mortality of approximately 4% [6].

The 2000 guideline by the ‘Status Epilepticus Working Party’ for treating and preventing status epilepticus by treating prolonged convulsive seizures (lasting more that 5 or 10 minutes) in children in the UK, advised infusion of 18 mg/kg of Phenytoin by slow intravenous (*iv*) infusion over 20 minutes as a third line treatment if other treatments (generally benzodiazepines) had failed to control the seizure [7]. However, there is little agreement between hospital protocols when treating CSE in children globally, and it is well known that many hospitals in the UK and in North America use 20 mg/kg dose [8].

The objective of this study was to test medical students, trainees and consultant doctors as part of an educational exercise in dose calculation, and see if it is easier and less prone to error to calculate a dose of 20 mg/kg rather than 18 mg/kg.

## Methods

A standard question paper was prepared, comprising five clinical scenarios with children of varying ages and estimated body weights. Medical students, trainee doctors at registrar and senior house officer (SHO) level, and consultant paediatricians were asked to complete the exercise, confidentially, anonymously, in private, as an educational exercise, by one of two medical students (SD, PS).

Calculations were done with and without a calculator, for 18 mg/kg and for 20 mg/kg in randomised order. Speed was recorded with a stop watch, and errors were determined. For our exercise, only calculation errors of greater than 10% different from the correct dose were counted as significant errors.

The whole exercise took 5–10 minutes of the student’s or doctor’s time.

The data analysis was performed using SPSS version 18 (SPSS Inc, Chicago, IL). Continuous variables were tested for normality using Kolmogrov-Smirnov test and Wilcoxson signed rank test was used for paired data when not normally distributed. One way ANOVA with *post hoc* analysis using Dunnett C test (unequal variance) was performed for normally distributed data when comparing effect of level of seniority on time taken to calculate. The categorical data was analysed using Fischer’s exact test and p values below 0.05 were taken as significant.

This was not experimental research, this was an educational exercise, so no ethics committee approval was required and no written consent was obtained,

## Results

Data was collected from the 20 scenarios as completed by 15 consultant paediatricians, 15 registrars, 15 SHOs, and 15 medical students. All answered all 20 scenarios, giving a total of 300 answers per doctor/student group, and 300 answers per type of calculation (Table 1).

**Table 1:** Number of dose calculation errors observed

	**18 mg/kg dose**		**20 mg/kg dose**		**Effect of dose**		**Effect of calculator use**	
	**Calculator used**	**No calculator**	**Calculator used**	**No calculator**	**Calculator used**	**No calculator**	**18 mg/kg dose**	**20 mg/kg dose**
Significant error >10%	2/300	28/300	3/300	1/300	NS	*	*	NS
Speed of calculation in seconds (range)	8 (2–36)	18 (2–77)	6 (1–39)	4 (1–44)	**	**	**	NS

Comparison of number of significant errors in drug calculation of > 10%, when asked to calculate 18 mg/kg dose and 20 mg/kg dose with and without the use of the calculator (top row). Speed of calculation (seconds) expressed as median and range for making the calculations when asked to calculate 18 mg/kg dose and 20 mg/kg dose with and without the use of a calculator (bottom row).

* p < 0.001 Fischer exact test.

** p < 0.001 Wilcoxson sign ranked test.

**Figure 1:**
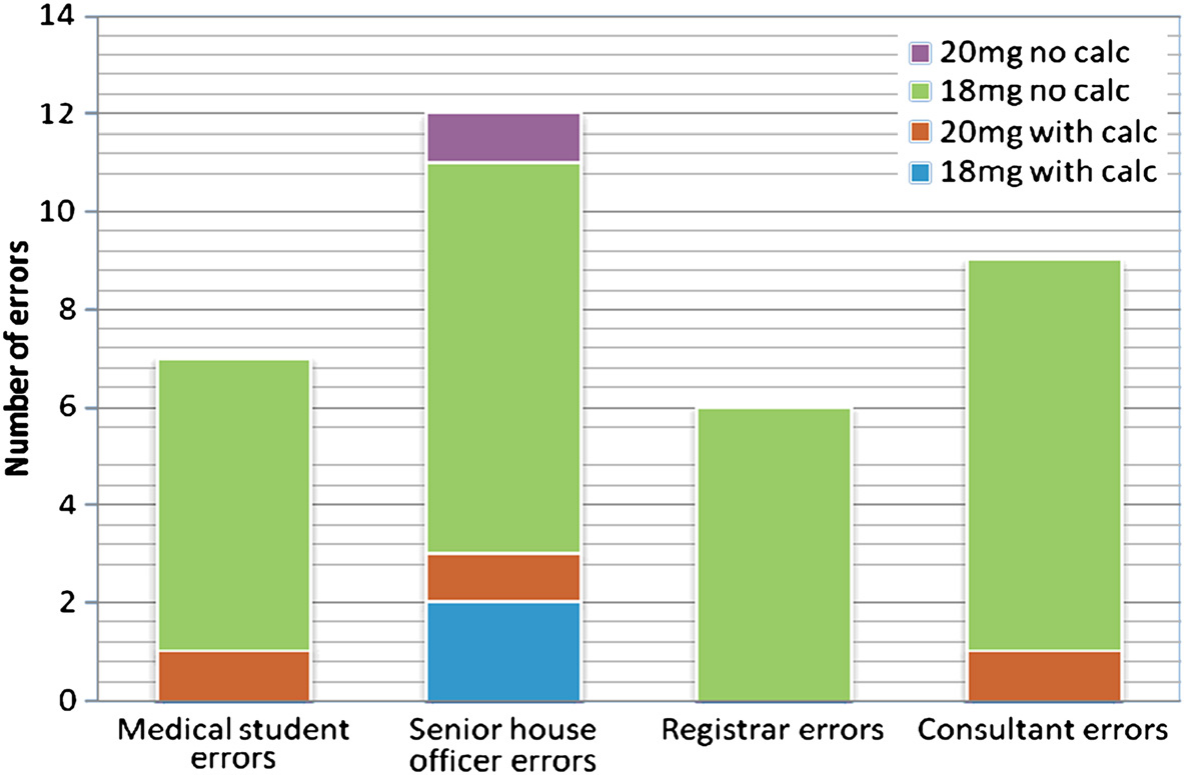
Significant errors by type of calculation and category of doctor or medical student.

**Figure 2:**
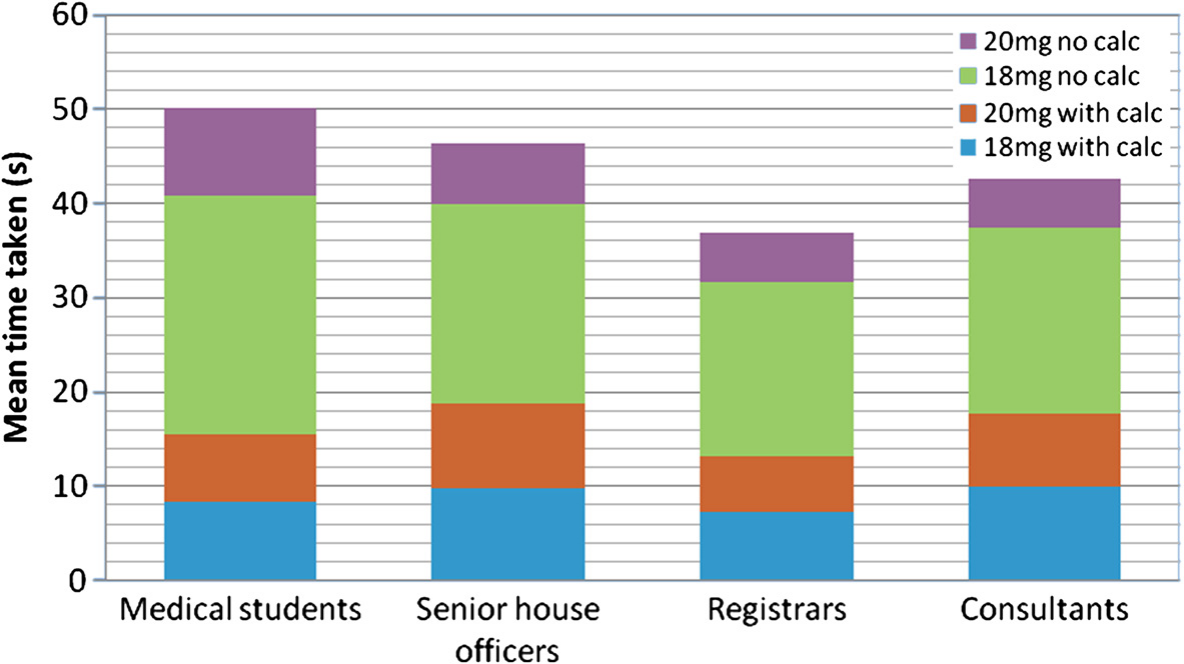
Mean time taken to perform the calculation and category of doctor or medical student.

**Figure 3:**
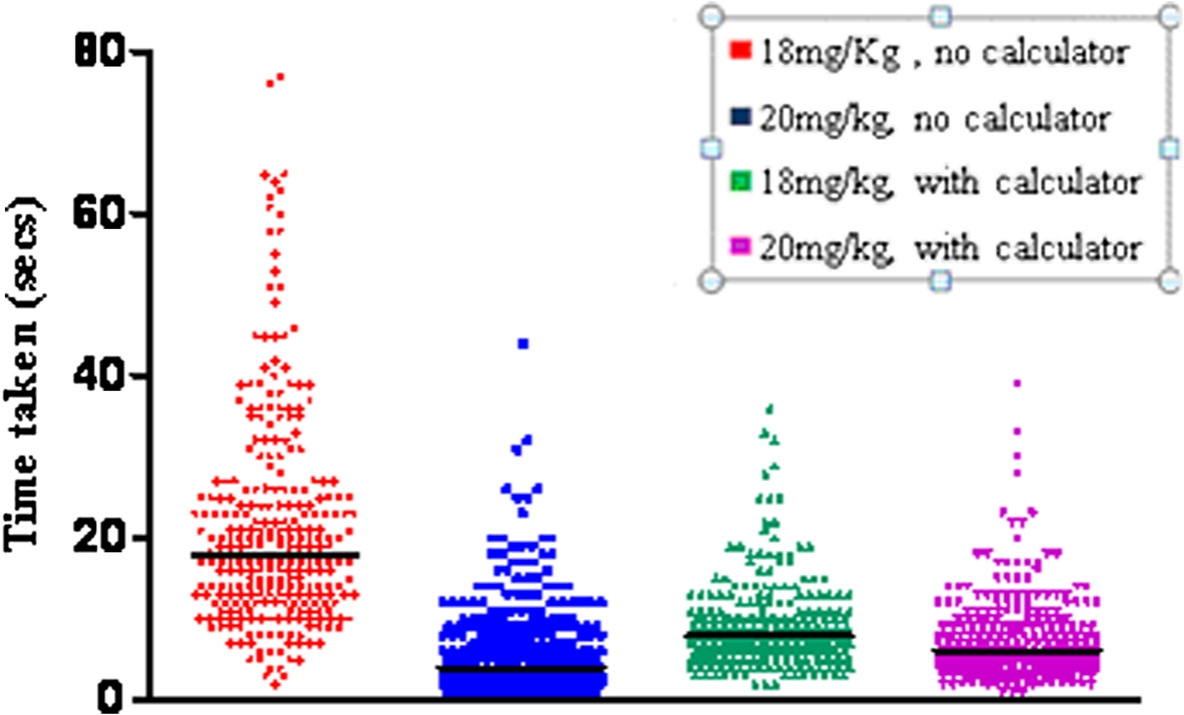
Scatter plot of time taken with and without a calculator to calculate 18 mg/kg and 20 mg/kg.

**Figure 4:**
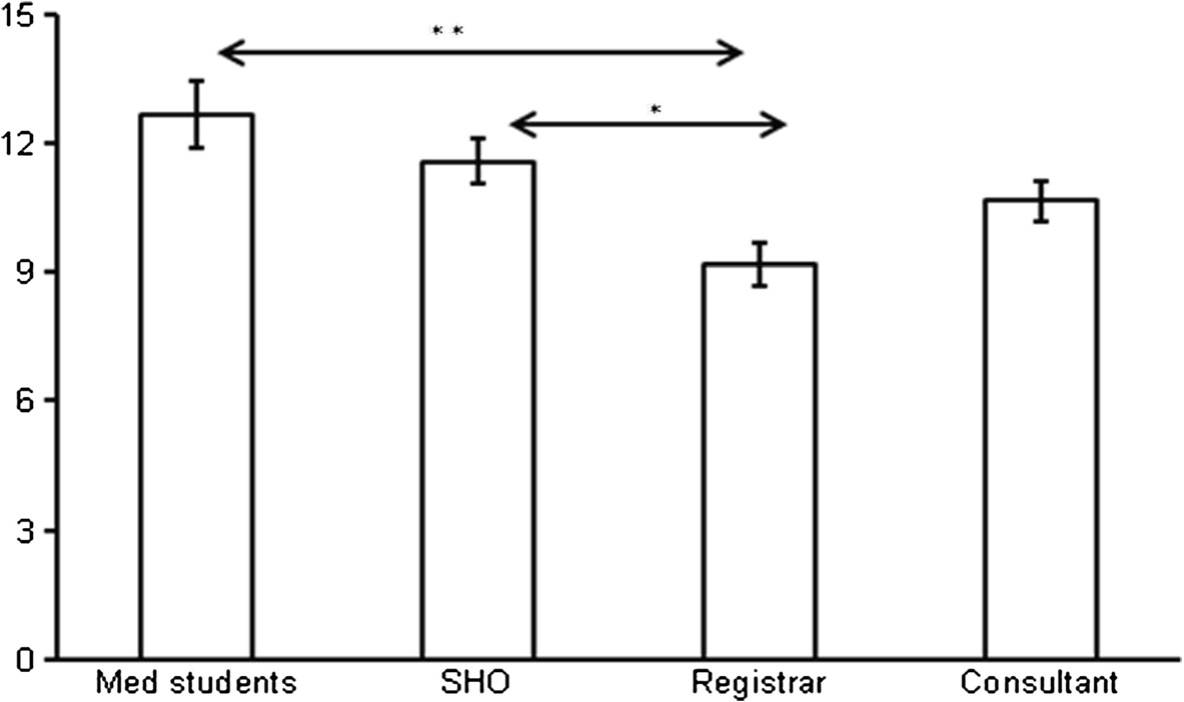
Time taken to perform the calculation by seniority.

The students’ and doctors’ performances were similar, with respect to the significant error rate (Figure 1). There was a significant effect of seniority on the time taken to calculate the dose (Figure 2, Figure 3), F (3,658) = p < 0.05. There was a significant quadratic trend with the time taken decreasing with seniority at registrar level and then again increasing with seniority, F(3, 658) = p = 0.028. *Post hoc* analysis shows registrars’ calculations were significantly faster than medical students’ (p < 0.001) and SHO’s (p = 0.006) (Figure 4). There was no significant order effect.

### Error rate > 10%

When comparing the 2 doses, the numbers of errors more than 10% were significantly less for the 20 mg/kg dose (0.33%) compared to the 18 mg/kg dose (9.3%) (p < 0.0001, Fischer exact test) when not using the calculator. The odds ratio for making a significant error is 30.77 with increased risk for the 18 mg/kg calculation. There was no significant difference in the significant error rate when using a calculator.

When using the 18 mg/kg dose, using a calculator significantly decreased the significant error rate from 9.3% to 0.66% (p < 0.001). The odds ratio of making an error while calculating the 18 mg/kg dose without a calculator was 15.38 as compared to when using a calculator.

For the 20 mg/kg doses, there was no difference in significant error rate with or without a calculator (1% *vs.* 0.3%), in fact, with the 20 mg/kg dose, the significant error rate was less *without* a calculator.

### Speed of calculation

When comparing the 2 doses, the time taken to perform the calculations was significantly decreased using the 20 mg/kg dose as compared to 18 mg/kg dose with (median 6 seconds, and 8 seconds respectively, T = 102, p < 0.001, r = 0. 21) or without (median 4 seconds, and 18 seconds respectively, T = 39, p < 0.0001, r = 0.79) a calculator.

For the 18 mg/kg doses, speed of calculation was better than halved by using a calculator (8.0 *vs.* 18.0 seconds) which is statistically significant (T = 39, p < 0.001, r = 0.75), however for the 20 mg/kg dose there was no significant difference in time taken with (median 6 seconds) or without (median 4 seconds) the calculator. In fact the speed was quicker without the calculator for the 20 mg/kg dose.

## Discussion

Medication errors are considered to be the commonest type of medical error, [9-11] and recent reviews have established that paediatric patients are at particularly high risk compared to adults [12,13]. It is estimated that the true incidence of paediatric dosing errors could be approximately 500,000 per year in England. There is, therefore, an urgent need to minimise such errors [14].

Published literature confirms that some healthcare professionals have difficulty calculating correct doses [15-17].

Phenytoin is one of the most effective drugs for treating acute convulsive seizures, whether primarily or secondarily generalised, and status epilepticus. The main advantage of Phenytoin is the relative lack of sedating effect. However, it is considered one of the medicines most commonly responsible for dosing errors in childhood by the Royal College Paediatrics and Child Health [18].

Historically, doses quoted for *iv* Phenytoin and Phenobarbitone range from 15–20 mg/kg. The guidelines already recommend 20 mg/kg as the dose for *iv* Phenobarbitone [7]. The difference between 20 mg/kg and 18 mg/kg, 2 mg/kg is 11.1% of 18 mg/kg. This is relatively small. The 18 mg/kg dose was first published to our knowledge in the paper by David M. Treiman *et al*[19]. This report does not justify their choice of 18 mg/kg over 20 mg/kg. We feel it implausible that such a small increase in the dose would have an overall pharmacological effect in this population, especially as the child’s weight is often estimated rather than measured as it is a medical emergency and they are convulsing, which makes weighing them impractical. We feel that a randomised controlled trial would be too costly and cumbersome for such a minor difference in dose, but that routine surveillance (clinical audit) would be useful to compare 18 mg/kg and 20 mg/kg as used in different units. At least major adverse events would be recorded this way.

We contacted Pfizer pharmaceutical company which now owns Parke-Davis who initially marketed EPANUTIN^®^ (phenytoin sodium). According to Pfizer the dose of 18 mg/kg quoted was derived from numerous clinical pharmacology studies (dose response studies) along with safety data from their phase 3 clinical programme.

Intravenous Phenytoin infusion can sometimes cause adverse cardiovascular effects, including bradycardia or hypotension [20]; hence in children the infusion rate should not exceed 1 mg/kg/min and should be administered with cardiac monitoring.

It is well known that many hospitals’ local guidelines advocate 20 mg/kg for *iv* Phenytoin for the management of children with prolonged seizures. To the best of our knowledge there have been no reports suggesting an increased risk of adverse effects with the 20 mg/kg dose compared with 18 mg/kg dose.

For this educational exercise, only calculation errors greater than 10% different from the correct dose were counted as significant errors.

The significant error rate was considerably lower and the speed much quicker for calculating 20 mg/kg dose when compared with the current recommended dose 18 mg/kg dose, without a calculator [7].

The exercise demonstrated that doctors and medical students will make errors in simple dose calculations in at best 0.3-1% of calculations even with a calculator. These errors may be higher in the real life scenarios as managing status epilepticus is a medical emergency which can be stressful, making us more prone for errors. This underlines the importance of checking all dose calculations. The longer time taken to calculate 18 mg/kg than 20 mg/kg reflects the increased difficulty of the calculation without an electronic calculator, and while many doctors and nurses in emergency health care settings have their personal calculators to hand, when a calculator is not immediately to hand, trying to find one will add to delays and stress. Furthermore, it is good practice when using a calculator to mentally check the result to see if it is “ball-park” correct, in case of a typing error, such as a misplaced decimal point that could lead to a ten-fold dosing error.

We propose that new status epilepticus guidelines should make an attempt to minimise *iv* Phenytoin dose calculation difficulties and minimise the risk of errors and therefore recommend 20 mg/kg.

In the latest edition of ‘Advanced Paediatric Life Support’, the status epilepticus algorithm now advises 20 mg/kg *iv* Phenytoin, replacing the previously recommended dose of 18 mg/kg [21].

## Conclusions

Medication errors are common and children are at particular risk. We recommend *iv* Phenytoin at 20 mg/kg rather than 18 mg/kg. This will make the calculation easier and reduce the risk of significant errors. Ease of dose calculation without an electronic calculator should be taken into account when making recommendations for drug doses in children, especially those to be used in emergency situations. All dose calculations should be checked.

## Competing interests

All the authors declare that they have no financial and no non-financial competing interests.

## Authors’ contributions

WPW and VS conceived and planned the excersise, PS and SD undertook the dose calculation educational exercises with the volunteers, collected the data and made preliminary analyses. VS, WPW, PS, SD completed the analyses and MP, SD and WPW undertook the literature review. All the authors contributed to drafting the paper. All authors read and approved the final manuscript.

## Pre-publication history

The pre-publication history for this paper can be accessed here:

http://www.biomedcentral.com/1471-2431/13/60/prepub

## What is known about this topic

1. Some guidelines recommend Phenytoin *iv* 18 mg/kg, some 20 mg/kg for prolonged convulsive epileptic seizures not responding to at least 2 doses of benzodiazepine.

2. A seminal paper by Treiman *et al.*, published in 1998 used *iv* Phenytoin 18 mg/kg.

## What this study adds

1. Students and prescribers were significantly less likely to make significant dose errors when calculating 20 mg/kg doses than 18 mg/kg doses, without an electronic calculator.

2. Medical students, trainees and consultants all made errors when calculating doses using 18 mg/kg without a calculator, in approximately 9% of their calculations.
